# Effects of 16 Weeks of Taekwondo Training on the Cerebral Blood Flow Velocity, Circulating Neurotransmitters, and Subjective Well-Being of Obese Postmenopausal Women

**DOI:** 10.3390/ijerph182010789

**Published:** 2021-10-14

**Authors:** Yong-Kuk Lee, Su-Youn Cho, Hee-Tae Roh

**Affiliations:** 1 Department of Taekwondo, College of Arts and Physical Education, Dong-A University, Busan 49315, Korea; DongaLee94@dau.ac.kr; 2 Exercise Physiology Laboratory, Department of Physical Education, Yonsei University, Seoul 03722, Korea; 3 Department of Taekwondo, Youngsan University, Yangsan-si 50510, Korea; 4 Department of Sports Science, College of Health Science, Sun Moon University, 70 Sunmoon-ro 221 beon-gil, Tangjeong-myeon, Asan-si 31460, Korea

**Keywords:** exercise training, body composition, mental health, cerebral blood flow, well-being

## Abstract

We investigated the effects of Taekwondo training on the body composition, serum lipid profiles, plasma neurotransmitter levels, cerebral blood flow velocities, and subjective well-being of 24 obese postmenopausal women. The women were randomly assigned into the experimental (*n* = 12) and control (*n* = 12) groups. The experimental group underwent Taekwondo training five times per week for 16 weeks, while the control group did not. All participants underwent evaluation for the following parameters before and after the intervention: body composition; serum lipid profiles; plasma serotonin and dopamine levels; cerebral blood flow velocities; positive and negative affect schedule (PANAS) scores; satisfaction with life scale (SWLS) scores. After the intervention, it was observed that the weight, body mass index, body fat percentage, total cholesterol, low-density lipoprotein cholesterol, and PANAS-NA (negative affect in the PANAS questionnaire) scores were significantly decreased (*p* < 0.05)—while the plasma serotonin levels were significantly increased (*p* < 0.05)—in the experimental group. Conversely, there were no significant changes in the cerebral blood flow velocities (*p* > 0.05). Taekwondo training can be effective in not only reducing obesity, but also in increasing the circulating neurotransmitters and enhancing the subjective well-being of obese postmenopausal women.

## 1. Introduction

The prevalence of obesity has increased worldwide, reaching pandemic levels over the past 50 years [1]. Obesity can substantially increase the risk of several diseases, such as type 2 diabetes, fatty liver disease, hypertension, myocardial infarction, and several cancers, ultimately leading to a decreased quality of life and life expectancy [1,2]. It has also been reported that obesity is involved in the development of psychiatric disorders, such as depression and anxiety, which threaten the mental health of women more severely than that of men [3,4]. A decrease in estrogen secretion after menopause may increase the risk of obesity due to increased body fat and can simultaneously induce depression and anxiety symptoms, particularly in menopausal women [5,6]. In addition, previous studies have suggested that obesity can reduce cerebral perfusion and blood flow velocity [7,8,9]. Specifically, Selim et al. reported that a high body mass index (BMI) was associated with a decreased cerebral blood flow (CBF) velocity and an increased cerebrovascular resistance [7]. A study by Knight et al. on older adults revealed that the CBF decreased with increasing age, and this decrease was significantly higher in women. Furthermore, they noted that increases in the BMI, waist-to-hip ratio (WHR), and waist size were associated with a reduction in the CBF; it was suggested that if the waist size increased by 1 cm, the corresponding decrease in the CBF was the same as that associated with one year of aging [8].

Several studies have already demonstrated that regular exercise and increases in the amount of physical activity are effective in preventing and managing obesity [10,11]. In addition, exercise can improve the mental health by alleviating symptoms of depression and anxiety through an increase in the secretion of neurotransmitters, such as serotonin and dopamine [12,13], and can also induce an increase in the CBF [8,14]. In a previous study by Knight et al., it was proposed that increasing the physical activity could halt the reduction in the CBF caused by obesity. Furthermore, Robertson et al. also reported that physical training could increase the CBF in stroke patients [14]. It has been reported that the positive effects of exercising can also be induced through Taekwondo [15,16,17,18]. Specifically, Fong and Ng critically reviewed 23 related papers to verify the effects of Taekwondo on physical fitness and noted that Taekwondo training effectively improved the body composition through fat loss and improved the aerobic capacity and flexibility [15]. Roh et al. also reported that 16 weeks of Taekwondo training effectively reduced the body weight and BMI in overweight and obese subjects [16]. Several previous studies have analyzed healthy children and adults using sub-scales for mood and have reported a significant reduction in the scores for tension, depression, and fatigue-inertia items following regular Taekwondo training [17,18]. This suggests that Taekwondo training may also be effective in promoting mental health through a yet-unclear mechanism.

As described above, obesity can cause various chronic diseases and impair the mental health, resulting in a negative impact on the subjective well-being (SWB). Regular Taekwondo training reduces obesity, enhances neurotransmitter secretion, and increases the CBF velocity, all of which positively affect the SWB. Furthermore, Lee et al. analyzed the average energy consumption when performing *Poomsae*, a unique training form of Taekwondo, and found that approximately 5.06 kcal/min of energy could be consumed; this suggests that Taekwondo training is an effective exercise type for promoting successful aging [19]. Furthermore, not only is Taekwondo a popular sport in Korea, but findings from several previous studies also demonstrate its greater effectiveness in improving the mental health and cognitive function as compared with other forms of exercise [18,20]. Bae and Roh reported on how Taekwondo could be more effective in improving the mood state and development of social skills as compared with other sports, such as bowling, table tennis, and golf [18]. Lakes et al. further suggested that compared with traditional physical education classes, physical education classes that involve Taekwondo may be more effective in improving the cognitive function [20]. The purpose of this study was to verify the effects of 16 weeks of Taekwondo training on the body composition, serum lipid profiles, plasma neurotransmitter levels, CBF velocities, and SWB in obese postmenopausal women.

## 2. Methods

### 2.1. Study Design and Participants

This study was designed as a single-blind, interventional, randomized controlled trial in Korea. The study participants comprised 24 obese postmenopausal women. The inclusion criteria were as follows: (1) postmenopausal women who did not menstruate (no menstruation for at least the last one year), (2) postmenopausal women who were obese (body fat percentage (BFP) > 32%; the criteria for obesity were determined according to previous studies [21,22]), (3) non-participation in any regular exercise program (defined as <20 min of exercise twice a week), and (4) no experience with Taekwondo training. Conversely, the exclusion criteria were as follows: (1) postmenopausal women who had neurological or psychiatric diseases and (2) postmenopausal women who were on diet-related medications. All participants were fully informed of the study procedure and a signed informed consent was obtained to this effect; this indicated that they understood the study procedure and the risks (potential pain when drawing blood) and benefits (a small monetary incentive) of participation. They were notified that withdrawal from participation would confer no negative consequences. The study protocol was approved by the institutional review board of Youngsan University (IRB No. YUSIRB-201610-BR-005-01), and the study conformed to the standards set by the latest revision of the Declaration of Helsinki. Using the G*Power software, the number of participants required was calculated to be 20 with an effect size (ES) of 0.40, α value of 0.05, and desired statistical power (1-β) of 0.90. However, we considered 24 participants after taking into account dropouts at a later stage. These participants were randomly assigned to the experimental group (*n* = 12), which participated in a 16-week Taekwondo training, and the control group (*n* = 12), which underwent no training for 16 weeks.

### 2.2. Study Procedure

The study procedure involved participant recruitment and selection, followed by testing before and after the 16-week intervention. Thirty applicants volunteered to partake in this study through a notice of recruitment. They were referred to the laboratory to undergo a survey that included body composition measurements in order to determine whether they met the inclusion criteria. Thereafter, the 24 selected study participants underwent a “pre-test” consisting of anthropometric measurements, blood tests, and measurements of the CBF velocity and the SWB. The participants underwent the same assessments in a “post-test” following the 16-week intervention period. All selected participants in the experimental group completed the 16-week intervention without attrition, and all 24 participants submitted to the pre- and post-tests.

### 2.3. Anthropometric Measurements

The anthropometric measurements included in the study were height and body composition. Height was measured to the nearest 0.1 cm using a stadiometer (SECA213; SECA, Hamburg, Germany), with the participant standing upright on bare feet. Body composition was measured using a body composition analyzer (Inbody720; Biospace, Seoul, Korea) after 12 h of fasting. The subjects donned short-sleeved tops and pants, and it was ensured that no metal object was in contact with the body before making the measurements.

### 2.4. Blood Sampling and Analysis

From each subject, 8 mL of blood was collected at rest from an antecubital vein using a 21-gauge needle after a 12-h fast. Half of the sample was collected in a serum separator tube, while the other half was collected in an ethylenediamine tetra-acetic acid tube. Blood separation was performed by centrifugation of the samples at 3000 rpm (2000× *g*) for 15 min. The serum total cholesterol (TC), triglyceride (TG), and high-density lipoprotein cholesterol (HDL-C) levels were determined via standard enzymatic colorimetric techniques using the CHOL2 (Roche Diagnostics GmbH, Mannheim, Germany), TRIGL (Roche Diagnostics GmbH, Mannheim, Germany), and HDL-Cholesterol Gen. 4 (Roche Diagnostics GmbH, Mannheim, Germany) assays, respectively. The low-density lipoprotein cholesterol (LDL-C) levels were calculated using the Friedewald formula [23]. The plasma serotonin and dopamine levels were analyzed through colorimetry using a Serotonin ELISA Kit (ab133053; Abcam, Cambridge, UK) and a Dopamine ELISA Kit (KA1887; Abnova, Taipei, Taiwan), respectively. The absorbances of serotonin and dopamine were observed at 405 and 450 nm, respectively, using a spectrophotometer (Tecan Sunrise; TECAN GmbH, Salzburg, Austria).

### 2.5. CBF Velocity Monitoring

The CBF velocity was assessed using the methods described by Aaslid et al. [24] and Cho et al. [25]. Measurements were taken using the transcranial Doppler system following 15 min of rest in a supine position. A 2.0-MHz probe was used to measure the peak-systolic (sFV: cm/s), end-diastolic (dFV: cm/s), and mean flow velocities (mFV: cm/s) in the middle cerebral artery (MCA) through the right temporal window. The pulsatility index (PI) was automatically calculated using the equation: PI = (sFV − dFV)/mFV.

### 2.6. SWB Measurements

The SWB was measured with the Positive and Negative Affect Schedule (PANAS) and the Satisfaction with Life Scale (SWLS) after referring to a previous study [26]. Lim et al. [27] developed and standardized a Korean version of the 20-item PANAS questionnaire developed by Watson et al. [28]. It consists of two factors, positive affect (PA) and negative affect (NA), with both factors carrying 10 items each; each item is rated on a five-point scale ranging from 1 (“very slightly or not at all”) to 5 (“extremely”). Possible PA and NA scores range from 10 to 50, with higher scores indicating a higher degree of PA or NA. The SWLS was developed by Diener et al. [29] as a tool for evaluating the overall life satisfaction [30]. This scale consists of five items in total, and each item is rated on a seven-point scale (1 = “strongly disagree” and 7 = “strongly agree”). Possible life satisfaction scores range from 5 to 35, with higher scores indicating higher levels of life satisfaction.

### 2.7. Taekwondo Training Protocol

Only the experimental group participated in the 16-week Taekwondo training sessions, while the control group continued with their daily lives without attending these sessions. The Taekwondo sessions were offered five times a week; each session was of 60 min (Taekwondo: 40 min; warm-up and cool-down exercises: 10 min). All participants wore a wireless heart-rate analyzer (Polar A5; Polar, Kempele, Finland) during the sessions. The sessions were designed based on a previous study by Cho and Roh [31], and consisted of *Poomsae* (Taegeuk 1~4 Jang), kicking and step exercises, and *Taekwon* aerobics, with an exercise intensity of 50–80% of the maximum heart rate. A Taekwondo expert instructor conducted all interventions through demonstration and coaching.

### 2.8. Statistical Analyses

Statistical analysis was performed using SPSS version 26.0 for Windows (IBM Corp., Armonk, NY, USA). Data were expressed as means ± standard deviation (SD) for all dependent variables. A two-way analysis of variance (ANOVA) was conducted to examine the differences in each dependent variable between the groups. Post hoc analysis was conducted using an independent *t*-test (intergroup differences within the same time period) and a paired *t*-test (intragroup differences between different time periods). The level of significance was set to 0.05.

## 3. Results

### 3.1. Characteristics of the Participants

The baseline characteristics of the participants are shown in Table 1; no variables differed significantly between the two groups (*p* > 0.05).

### 3.2. Changes in the Body Composition after the 16-Week Intervention

Post-intervention changes in the body composition of the experimental and control groups are shown in Table 2. The two-way ANOVA revealed significant differences after 16 weeks (group × time interaction) for the weight (*F* = 8.108, *p* = 0.009), BMI (*F* = 8.374, *p* = 0.008), and BFP (*F* = 13.614, *p* = 0.001). Post hoc test results revealed a significant decrease in the weight, BMI, and BFP in the experimental group, but not in the control group, after the intervention (*p* < 0.05). There were no significant differences in the pre- and post-intervention skeletal muscle mass (*F* = 2.117, *p* = 0.160) in either group.

### 3.3. Changes in the Serum Lipid Profiles after the 16-Week Intervention

Post-intervention changes in the serum lipid profiles of the experimental and control groups are shown in Table 3. The two-way ANOVA revealed significant differences after 16 weeks (group × time interaction) for the TC (*F* = 4.922, *p* = 0.037) and LDL-C (*F* = 4.761, *p* = 0.040). Post hoc test results revealed significant decreases in the TC and LDL-C in the experimental group (*p* < 0.05), but not in the control group, after the intervention. There were no significant differences in the pre- and post-intervention TG (*F* = 0.280, *p* = 0.602) and HDL-C (*F* = 0.250, *p* = 0.622) in either group.

### 3.4. Changes in the CBF Velocities after the 16-Week Intervention

**Figure 1:**
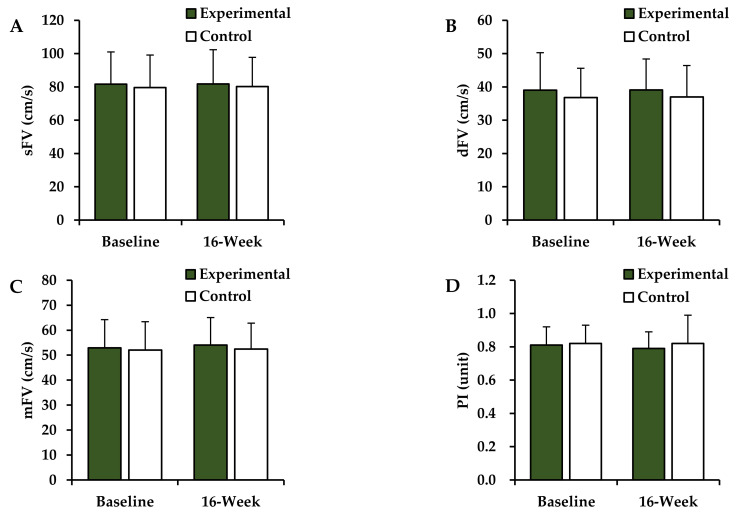
Changes in CBF velocities. Values are presented as mean ± SD. (**A**) sFV, systolic flow velocity; (**B**) dFV, diastolic flow velocity; (**C**) mFV, mean flow velocity; (**D**) PI, pulsatility index.

Post-intervention changes in the CBF velocities of the experimental and control groups are shown in Figure 1. The two-way ANOVA revealed no significant differences after 16 weeks (group × time interaction) for the sFV (*F* = 0.054, *p* = 0.818), dFV (*F* = 0.005, *p* = 0.944), mFV (*F* = 0.149, *p* = 0.703), and PI (*F* = 1.046, *p* = 0.318).

### 3.5. Changes in the Plasma Neurotransmitter Levels after the 16-Week Intervention

**Figure 2:**
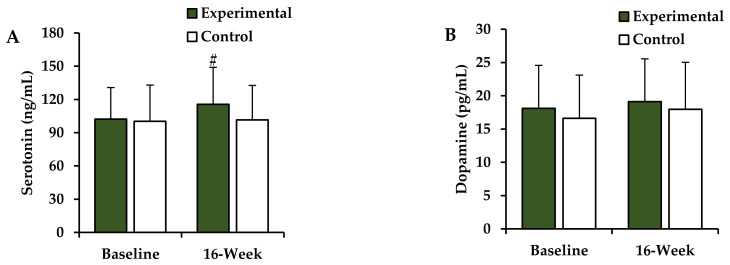
Changes in plasma neurotransmitters. Values are presented as mean ± SD. (**A**) serotonin; (**B**) dopamine; ^#^ Compared with baseline values within the experimental group (*p* < 0.05).

Post-intervention changes in the plasma neurotransmitter levels of the experimental and control groups are shown in Figure 2. The two-way ANOVA revealed a significant difference after 16 weeks (group × time interaction) for the plasma serotonin levels (*F* = 4.981, *p* = 0.036). Post hoc test results revealed significant increases in the plasma serotonin levels in the experimental group (*p* < 0.05), but not in the control group, after the intervention. There were no significant differences in the pre- and post-intervention plasma dopamine levels (*F* = 0.062, *p* = 0.806) in either group.

### 3.6. Changes in the SWB Indices after the 16-Week Intervention

**Figure 3:**
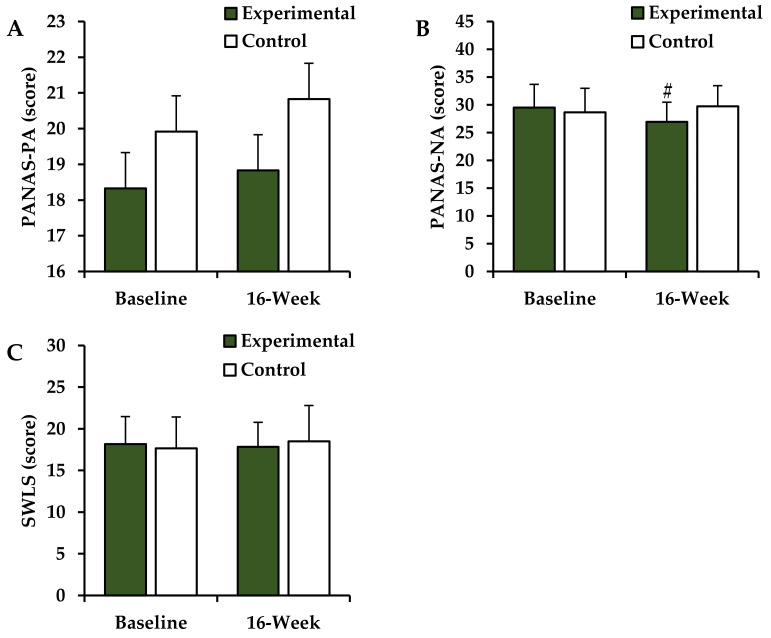
Changes in SWB indices. Values are presented as mean ± SD. (**A**) PANAS-PA, positive affect in the Positive and Negative Affect Schedule questionnaire; (**B**) PANAS-NA, negative affect in the Positive and Negative Affect Schedule questionnaire; (**C**) SWLS, Satisfaction with Life Scale; ^#^ Compared with baseline values within the experimental group (*p* < 0.05).

Post-intervention changes in the SWB indices of the experimental and control groups are shown in Figure 3. The two-way ANOVA revealed a significant difference after 16 weeks (group × time interaction) for the PANAS-NA score (*F* = 9.576, *p* = 0.005). Post hoc test results revealed significant decreases in the PANAS-NA scores in the experimental group, but not in the control group, after the intervention (*p* < 0.05). There were no significant differences in the pre- and post-intervention PANAS-PA (*F* = 0.188, *p* = 0.669) and the SWLS (*F* = 1.321, *p* = 0.321) scores in either group.

## 4. Discussion

This study demonstrated that Taekwondo training can effectively regulate the secretion of neurotransmitters and enhance the SWB in addition to improving the body composition and serum lipid profiles in obese postmenopausal women.

Taekwondo, a martial art that originated in Korea, is a popular sport worldwide and is also an official Olympic sport [25,32]. Several previous studies [15,16,25,31,33,34] have demonstrated the health benefits of regular Taekwondo training. Specifically, Taekwondo training can improve the physical strength, cardiovascular endurance, muscular strength, muscular endurance, and flexibility [15,16,25,33], in addition to promoting the development of brain function in growing children [25,34]. Positive effects have also been reported on the cognitive function in the elderly [31]. In addition, Taekwondo training has been reported to improve sociality and the state of mood [17,18]. In the present study, body composition and the serum lipid profiles in obese postmenopausal women were analyzed to verify the obesity reduction resulting from Taekwondo training; the body weight, BMI, and BFP were significantly decreased after 16 weeks of Taekwondo training. These results support those of previous studies [15,16] that reported significant improvements in the body composition following Taekwondo training; therefore, this suggests that the Taekwondo training program conducted in this study was sufficient for inducing weight loss. Fong and Ng suggested that Taekwondo training effectively improves the body composition by inducing fat loss [15], and Roh et al. reported a significant decrease in the body weight and BMI after 16 weeks of Taekwondo training [16]. Moreover, the American College of Sports Medicine recommends more than 225 min of exercise per week for weight loss [35], a recommendation complied with by the present study wherein an intervention with 300 min of Taekwondo training per week was performed.

Dyslipidemia refers to abnormal blood lipid levels and is defined by elevated TC, TG, and LDL-C levels and low HDL-C levels in the blood [35]. The causes of dyslipidemia include bad eating habits, lifestyle factors, and genetic factors, and regular exercise has been proven to be effective in regulating the blood lipid levels [35,36]. In this study, the TC and LDL-C levels decreased significantly after Taekwondo training, which may have been the main factor underlying the reduction in the BMI and BFP. In other words, dyslipidemia is a common comorbidity caused by an overweight state or obesity; excess body fat is associated with dyslipidemia development and positive correlations have been reported between the BMI, TC, and LDL-C [37,38,39]. Conversely, no significant changes were observed in the TG and HDL-C levels in this study. Thus, we hypothesize that although changes in the body mass and fat mass are associated with an improvement in the blood lipid profiles, not all changes are consistent. Based on previous research findings that indicate that such improvements require a minimum exercise threshold and may be dependent on the type of training [35,40], there may be a need to verify the above-mentioned hypothesis using different types of training and/or intensities.

Obesity can cause mood disorders, such as anxiety and depression, which, in turn, can lead to an increase in obesity; thus, the effect is bidirectional [41]. Chronic low-grade inflammation and dysregulation of monoamine neurotransmitters, such as serotonin, dopamine, and norepinephrine, are representative mechanisms of mental health inhibition due to obesity [41,42]. Kim et al. used principal component analysis to verify the plasma serotonin and dopamine levels and proposed that they may differ between the obese and normal models [42]. The present study analyzed the plasma serotonin and dopamine levels to verify the effects of Taekwondo training on neurotransmitters in obese postmenopausal women, and the results revealed that the plasma serotonin level was significantly increased after 16 weeks of training. These results suggest that Taekwondo training may regulate the secretion of neurotransmitters and play a significant role in their improvement through obesity reduction. Kim et al. found that compared with the normal model, the plasma serotonin level was significantly reduced in the obesity model induced by a high-fat diet; they reported a negative correlation between body weight and the plasma serotonin level [42].

Obesity has been proposed as one of the main causes of the age-related decrease in the CBF velocity. In addition, parameters such as the BMI, WHR, and waist size have been reported to have a negative correlation with the CBF velocity [7,8]. The decrease in the CBF velocity is not only observed in depression, but is also associated with several other neurological disorders, including Alzheimer’s disease [43,44]. It has been reported that increasing the amount of physical activity can alleviate the decrease in the CBF velocity due to obesity [8] and that regular aerobic-endurance exercise is associated with a high blood flow velocity in the MCA [45]. In the present study, the sFV, dFV, and mFV in the MCA were measured, and the PI was calculated to verify the effect of Taekwondo training on the CBF velocity in obese postmenopausal women. No significant differences in these parameters were noted despite an improvement in the obesity after 16 weeks of training. These results support those of previous studies [25,31] that did not identify changes in the CBF velocity following Taekwondo training as well. One of the drawbacks of this study is that we measured the CBF velocity only in the right temporal window after 16 weeks of training. However, Ainslie et al. suggested that a two-year exercise period would be required to improve the CBF velocity [45]. Akazawa et al. also measured the CBF velocities in both the left and right MCAs in postmenopausal women and calculated their mean; they reported a significant increase in the CBF velocity after aerobic exercise training [46].

It has been reported in a few previous studies that the benefits of participating in Taekwondo training are not only limited to physical aspects, but also to an improved holistic health through the promotion of the overall well-being of the body, mind, and spirit [18,47]. These studies verified the effects of Taekwondo training on the SWB in obese postmenopausal women using the PANAS and SWLS. No significant difference was noted in the PANAS-PA score after Taekwondo training, but the PANAS-NA score decreased significantly. These results suggest that Taekwondo training may effectively enhance the SWB, and changes in the blood neurotransmitter levels due to the training are believed to play a major role in this enhancement [48]. It has been reported that the biological PA is regulated by dopamine, while the NA is regulated by serotonin [49,50]. In fact, in a previous study by Depue et al., an increase in the dopamine levels had an influence on the PA, but not on the NA [49], and Knutson et al. reported that an increase in the serotonin levels could reduce the NA without changing the PA [50].

The limitations of this study should be addressed. First, the study participants were limited to obese women aged from 50 to 64 years. Second, although the study participants were postmenopausal women, their levels of sex hormones, such as estrogen, were not analyzed. Therefore, further studies are needed to verify the results obtained in this study.

## 5. Conclusions

Participation in Taekwondo training can lead to improved body composition and positive changes in the blood lipid levels and can positively affect mental health by regulating the secretion of neurotransmitters, such as serotonin. Thus, Taekwondo training can be recommended to obese postmenopausal women as a form of exercise to promote their well-being.

## Figures and Tables

**Table 1:** Characteristics of the participants.

Variable	Experimental (*n* = 12)	Control (*n* = 12)	*p*
Age (years)	56.0 ± 2.9	57.5 ± 2.9	0.223
Height (cm)	157.4 ± 4.7	156.6 ± 3.3	0.620
Weight (kg)	64.0 ± 5.8	62.6 ± 6.2	0.576
BMI (kg/m^2^)	25.8 ± 2.0	25.5 ± 1.7	0.674
SMM (kg)	22.0 ± 2.7	20.8 ± 2.5	0.288
BFP (%)	37.0 ± 3.1	36.3 ± 2.6	0.630
TC (mg/dL)	183.6 ± 42.8	184.9 ± 42.7	0.939
TG (mg/dL)	127.6 ± 53.1	132.5 ± 42.4	0.805
LDL-C (mg/dL)	100.8 ± 38.8	102.8 ± 41.0	0.903
HDL-C (mg/dL)	53.6 ± 15.2	53.8 ± 15.2	0.979
sFV (cm/s)	81.7 ± 19.6	79.6 ± 19.3	0.796
dFV (cm/s)	39.0 ± 11.3	36.8 ± 8.8	0.605
mFV (cm/s)	52.9 ± 11.3	52.0 ± 11.4	0.845
PI (unit)	0.8 ± 0.1	0.8 ± 0.1	0.836
Serotonin (ng/mL)	102.2 ± 28.6	100.3 ± 32.7	0.883
Dopamine (pg/mL)	18.1 ± 6.5	16.6 ± 6.5	0.574
PANAS-PA (score)	18.3 ± 3.8	19.9 ± 4.2	0.340
PANAS-NA (score)	29.5 ± 4.2	28.7 ± 4.3	0.321
SWLS (score)	18.2 ± 3.3	17.7 ± 3.8	0.732

Values are presented as mean ± SD. BMI, body mass index; SMM, skeletal muscle mass; BFP, body fat percentage; TC, total cholesterol; TG, triglyceride; LDL-C, low-density lipoprotein cholesterol; HDL-C, high-density lipoprotein cholesterol; sFV, systolic flow velocity; dFV, diastolic flow velocity; mFV, mean flow velocity; PI, pulsatility index; PANAS-PA, positive affect in Positive and Negative Affect Schedule questionnaire; PANAS-NA, negative affect in Positive and Negative Affect Schedule questionnaire; SWLS, Satisfaction with Life Scale.

**Table 2:** Changes in body composition.

Variables	Experimental (*n* = 12)		Control (*n* = 12)		Interaction (Group × Time)	
	Baseline	16-Week	Baseline	16-Week	*F*	*p*
Weight (kg)	64.0 ± 5.8	60.1 ± 4.3 ^#^	62.6 ± 6.2	62.4 ± 5.1	8.108	0.009 **
BMI (kg/m^2^)	25.8 ± 2.0	24.3 ± 1.7 ^#^	25.5 ± 1.7	25.4 ± 1.3	8.374	0.008 **
SMM (kg)	22.0 ± 2.7	21.2 ± 2.2	20.8 ± 2.5	20.5 ± 2.1	2.117	0.160
BFP (%)	37.0 ± 3.1	33.8 ± 4.9 ^#^	36.3 ± 2.6	36.5 ± 2.2	13.226	0.001 **

Values are presented as mean ± SD. BMI, body mass index; SMM, skeletal muscle mass; BFP, body fat percentage; ^#^ Compared with baseline values within the experimental group (*p* < 0.05); ** *p* < 0.01.

**Table 3:** Changes in lipid profiles.

Variables	Experimental (*n* = 12)		Control (*n* = 12)		Interaction (Group × Time)	
	Baseline	16-Week	Baseline	16-Week	*F*	*p*
TC (mg/dL)	183.6 ± 42.8	169.8 ± 42.2 ^#^	184.9 ± 42.7	190.7 ± 42.5	4.922	0.037 *
TG (mg/dL)	127.6 ± 53.1	118.3 ± 42.5	132.5 ± 42.4	127.8 ± 32.6	0.280	0.602
LDL-C (mg/dL)	100.8 ± 38.8	93.5 ± 35.7 ^#^	102.8 ± 41.0	110.2 ± 40.6	4.761	0.040 *
HDL-C (mg/dL)	53.6 ± 15.2	57.8 ± 12.0	53.8 ± 15.2	55.9 ± 12.8	0.250	0.622

Values are presented as mean ± SD. TC, total cholesterol; TG, triglyceride; LDL-C, low-density lipoprotein cholesterol; HDL-C, high-density lipoprotein cholesterol; ^#^ Compared with baseline values within the experimental group (*p* < 0.05); * *p* < 0.05.

## Data Availability

Data generated and analyzed during this study are included in this article. Additional data are available from the corresponding author on request.
